# OLDER ADULTS’ ACCEPTANCE OF TELEGRACE: A HYBRID TELEHEALTH CARE MODEL

**DOI:** 10.1093/geroni/igad104.2312

**Published:** 2023-12-21

**Authors:** Alaina Preddie, Lauren Penney, Ashley Schwartzkopf, Cathy Schubert, Dawn Bravata, Teresa Damush

**Affiliations:** Richard L Roudebush VA Medical Center, Indianapolis, Indiana, United States; National Institutes of Health, Bethesda, Maryland, United States; Department of Veterans Affairs (VA), Indianapolis, Indiana, United States; Richard L Roudebush VA Medical Center, Indianapolis, Indiana, United States; Richard L Roudebush VA Medical Center, Indianapolis, Indiana, United States; Richard L Roudebush VA Medical Center, Indianapolis, Indiana, United States

## Abstract

The VA Geriatric Resources for Assessment and Care of Elders (GRACE) at Veterans Health Indiana is an interdisciplinary, home-based program providing comprehensive geriatric assessment and ongoing care management for community-dwelling older Veterans’ experiencing geriatric syndromes. TeleGRACE is an extension of the GRACE program and delivers care via a hybrid, telehealth home visit. The TeleGRACE technician operates the audio-visual equipment in the patient’s home while the clinical dyad (GRACE nurse practitioner and social worker) conducts the assessment. The specific aim of this study was to evaluate patient acceptability of TeleGRACE. We conducted a mixed methods study which included Likert-scale ratings and open-ended interviews. Participants used a 5-point scale to indicate agreement (5) to disagreement (1) regarding the acceptability of program elements ratings including ease with technician and virtual dyads; access; and clinical telepresence. The sample included 12 Veterans (mean [M] age, 78.7 years; standard deviation [SD] 7.5) who completed both an initial and follow-up TeleGRACE visit. Veterans reported high agreement of TeleGRACE acceptability (M=4.9, SD=0.3). They agreed: TeleGRACE hybrid visits helped them get care that they could not access otherwise (M=4.7, SD=0.65), they were as well taken care of by TeleGRACE virtual clinicians as those met at in-person visits (M=4.8, SD=0.4), they could hear (M=4.8, SD=0.6) and see (M=5.0, SD=0) the clinical dyads through the video visits, and they were able to ask direct questions of the TeleGRACE clinical staff (M=5.0, SD=0). These results indicated that TeleGRACE’s key elements of a hybrid telehealth home visit were acceptable to older, community-dwelling Veterans.

